# Equisetin Restores Colistin Sensitivity against Multi-Drug Resistant Gram-Negative Bacteria

**DOI:** 10.3390/antibiotics10101263

**Published:** 2021-10-18

**Authors:** Qi Zhang, Shang Chen, Xiaojia Liu, Wenhan Lin, Kui Zhu

**Affiliations:** 1 National Center for Veterinary Drug Safety Evaluation, College of Veterinary Medicine, China Agricultural University, Beijing 100193, China; zhangqivet@cau.edu.cn (Q.Z.); schen94@cau.edu.cn (S.C.); b20203050368@cau.edu.cn (X.L.); 2 State Key Laboratory of Natural and Biomimetic Drugs, Peking University, Beijing 100191, China

**Keywords:** equisetin, colistin, Gram-negative bacteria, drug combination, antimicrobial resistance

## Abstract

The overuse of antibiotics and the scarcity of new drugs have led to a serious antimicrobial resistance crisis, especially for multi-drug resistant (MDR) Gram-negative bacteria. In the present study, we investigated the antimicrobial activity of a marine antibiotic equisetin in combination with colistin against Gram-negative bacteria and explored the mechanisms of synergistic activity. We tested the synergistic effect of equisetin in combination with colistin on 23 clinical *mcr-1* positive isolates and found that 4 µg/mL equisetin combined with 1 µg/mL colistin showed 100% inhibition. Consistently, equisetin restored the sensitivity of 10 species of *mcr-1* positive Gram-negative bacteria to colistin. The combination of equisetin and colistin quickly killed 99.9% bacteria in one hour in time-kill assays. We found that colistin promoted intracellular accumulation of equisetin in colistin-resistant *E. coli* based on LC-MS/MS analysis. Interestingly, equisetin boosted ROS accumulation in *E. coli* in the presence of colistin. Moreover, we found that equisetin and colistin lost the synergistic effect in two LPS-deficient *A. baumannii* strains. These findings suggest that colistin destroys the hydrophobic barrier of Gram-negative bacteria, facilitating equisetin to enter the cell and exert its antibacterial effect. Lastly, equisetin restored the activity of colistin in a *G. mellonella* larvae infection model. Collectively, these results reveal that equisetin can potentiate colistin activity against MDR Gram-negative bacteria including colistin-resistant strains, providing an alternative approach to address Gram-negative pathogens associated with infections in clinics.

## 1. Introduction

The alarming emergence and dissemination of antimicrobial resistance constitute a crisis to One Health. It is predicted that antimicrobial resistance will lead to the death of 10 million people/year by 2050 [1]. The overuse and misuse of antibiotics and the scarcity of new drugs result in the migration of multidrug-resistant (MDR) bacteria from hospitals to the community. In 2017, the World Health Organization (WHO) introduced a list of “critical priority” pathogens—including the “ESKAPE” pathogens (*Enterococcus faecium*, *Staphylococcus aureus*, *Klebsiella pneumoniae*, *Acinetobacter baumannii*, *Pseudomonas aeruginosa* and *Enterobacter* species)—responsible for a global health problem [2]. Compared to Gram-positive bacteria, Gram-negative bacteria (*Escherichia coli*, *K. pneumoniae*, *P. aeruginosa* and *A. baumannii*) are among the primary infectious agents, owing to their dual-membrane envelope, which prevent many antibiotics from accessing targets. Therefore, future development strategies should pay more attention to antibiotics that are active against MDR Gram-negative bacteria. There was almost no novel compounds discovered that were active against Gram-negative bacteria in the past fifty years [3]. Thus, it is imperative to explore novel antibiotics or other approaches against MDR Gram-negative bacteria-associated infections. Otherwise, we will probably experience a pandemic that is similar to the current COVID-19 crisis [4,5].

The discovery of antibiotics against Gram-negative bacteria has been much slower due to the presence of an intrinsic outer membrane (OM) [6]. The OM is an asymmetric lipid bilayer consisting of an outer leaflet of lipopolysaccharide (LPS) and an inner leaflet of phospholipids. The LPS prevents an influx of large or hydrophobic antibiotics, such as vancomycin and erythromycin, restricting their access to intracellular targets [7]. Polymyxins are regarded as the last-resort antibiotics to treat MDR Gram-negative pathogens [8] and play a critical role in the era of antibiotic resistance [9], but the serious nephrotoxicity and neurotoxicity also limit their clinical use. The revival of colistin in recent years is largely due to the lack of novel antibiotics that are effective against the increasingly prevalent MDR Gram-negative infections, particularly the carbapenem-resistant Enterobacteriaceae. However, bacteria harboring an *mcr-1* plasmid-borne colistin resistance contribute to the rapid dissemination and ubiquitous distribution of colistin resistance [10,11]. Lacking the discovery of new drugs, the utilization of currently available drugs or the expansion of the antibacterial spectra would contribute to our arsenal against MDR Gram-negative bacteria [12]. For example, the development of advanced drug-delivery systems for antibacterial therapies [13] and the discovery of novel antibiotic adjuvants are becoming promising strategies to revitalize the existing antibiotics [14]. 

We recently found that a marine fungal symbiont efficiently produces equisetin with broad-spectrum antibacterial activities against MDR Gram-positive pathogens, including methicillin-resistant *Staphylococcus aureus* (MRSA) and vancomycin-resistant *Enterococci* (VRE) [15]. However, equisetin alone is ineffective against Gram-negative bacteria, probably due to the presence of OM barriers. Interestingly, equisetin as an adjuvant restores colistin sensitivity in colistin-resistant Gram-negative pathogens although the underlying mechanism remains unclear. In this study, we found that low levels of colistin combined with equisetin was effective against the 10 species of colistin-resistant Gram-negative bacteria that were tested, which, in turn, reduced the therapeutic toxicity of colistin. The combination of equisetin and colistin boosts the accumulation of ROS in *E. coli*. These results indicate that colistin destroys the membrane structure of Gram-negative bacteria, facilitating equisetin to enter the cell and exert the antibacterial effect.

## 2. Results

### 2.1. Equisetin Synergies with Colistin against MDR Gram-Negative Bacteria

**Figure 1:**
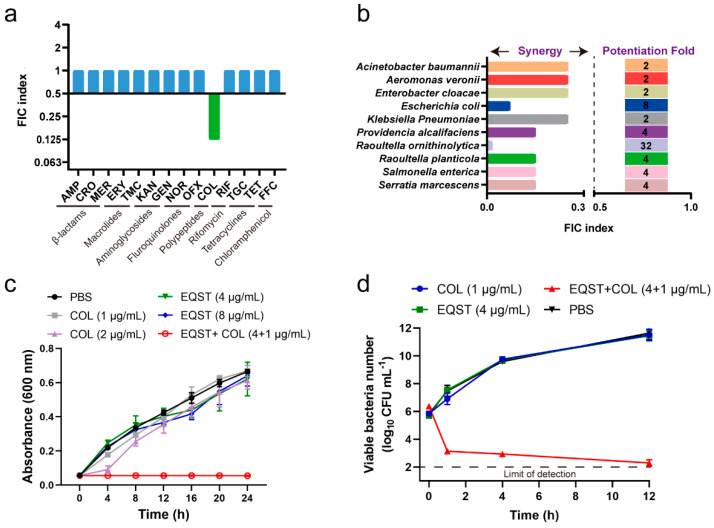
Equisetin restores colistin sensitivity against MDR Gram-negative bacteria. (**a**) The FIC indexes of antibiotics combined with equisetin against *E. coli* B2. Synergy is defined as an FIC of ≤0.5. AMP: ampicillin, CRO: ceftriaxone; MER: meropenem, ERY: erythromycin, TMC: tilmicosin, KAN: kanamycin, GEN: gentamycin, OFX: ofloxacin, NOR: norfloxacin, COL: colistin, RIF: rifampicin, TGC: tigecycline, TET: tetracyclines, FFC: florfenicol. (**b**) Synergistic antibacterial activities between equisetin and colistin against 10 species of *mcr-1* positive Gram-negative bacteria. FIC index ≤ 0.5, synergistic. (**c**) Growth curve of *E. coli* B2 after treatment with different combinations of equisetin and colistin. EQST: equisetin. (**d**) Time-dependent killing of *E. coli* B2 treated with equisetin combined with colistin. Data are representative of three independent experiments, mean ± s.d.

To expand the antibacterial spectrum of equisetin and to improve the efficiency of currently available antibiotics, we systematically assessed the synergistic activity between equisetin and other major classes of antibiotics against *E. coli* B2 (NDM-5 + MCR-1). We found that equisetin had no synergistic activity with other antibiotics except colistin, as was previously reported [15] (Figure 1a, Table S1). Then, we focused on the synergistic effect of equisetin and colistin against colistin-resistant bacteria. We tested the synergistic antibacterial effect of equisetin and colistin on 23 clinical *mcr-1* positive resistant isolates at different concentrations and found that equisetin (4 µg/mL) combined with colistin (1 µg/mL) inhibited 100% of the tested bacteria (Table 1 and Table S1). Consistently, equisetin restored colistin sensitivity in 10 species of *mcr-1* positive Gram-negative bacteria in the presence of equisetin (4 µg/mL) plus colistin (1 µg/mL) (Figure 1b, FIC index ≤ 0.5), which is consistent with our previous report [15]. Neither equisetin alone (4 µg/mL), nor sublethal levels of colistin (1 µg/mL, 1/8 MIC), affected the growth of *E. coli* (Figure 1c) but their combination sharply inhibited the growth of *E. coli*. Furthermore, the time-killing curve showed that equisetin combined with colistin rapidly killed more than 99% of *E. coli* within 1 h (Figure 1d). 

### 2.2. Mechanism of Equisetin Combined with Colistin against Bacteria

**Figure 2:**
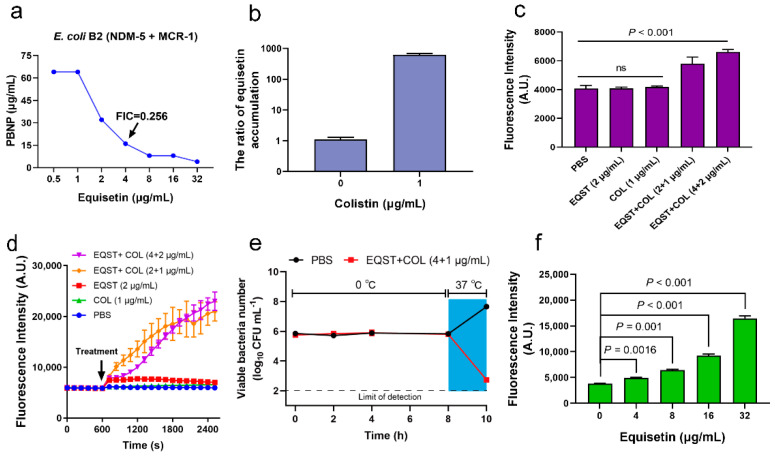
Mechanism of equisetin in combination with colistin against bacteria. (**a**) Synergistic antibacterial activities between equisetin and PBNP against *E. coli* B2. (**b**) Colistin promotes the accumulation of equisetin in bacterial cells. (**c**) Increased permeability of the outer membrane of *E. coli* B2 treated with drug combination, probed with NPN. (**d**) Increased permeability of the cytoplasmic membrane of *E. coli* B2 treated with drug combination, probed with PI. (**e**) The synergistic activity of equisetin and colistin is dependent on bacterial metabolic states. *E. coli* B2 cells at the exponential phase were incubated in the presence of EQST + COL (4 + 1 µg/mL) in ice-water mixtures (0 °C, 0–8 h) and culture tubes at 37 °C (8–10 h). (**f**) In the presence of 1 µg/mL colistin, the higher the concentration of equisetin, the more ROS accumulated in *E. coli*. Data are representative of three independent experiments and the mean of three biological replicates is shown. Error bars represent the s.d. and *p* values were determined using an unpaired, two-tailed Student’s *t*-test.

**Figure 3:**
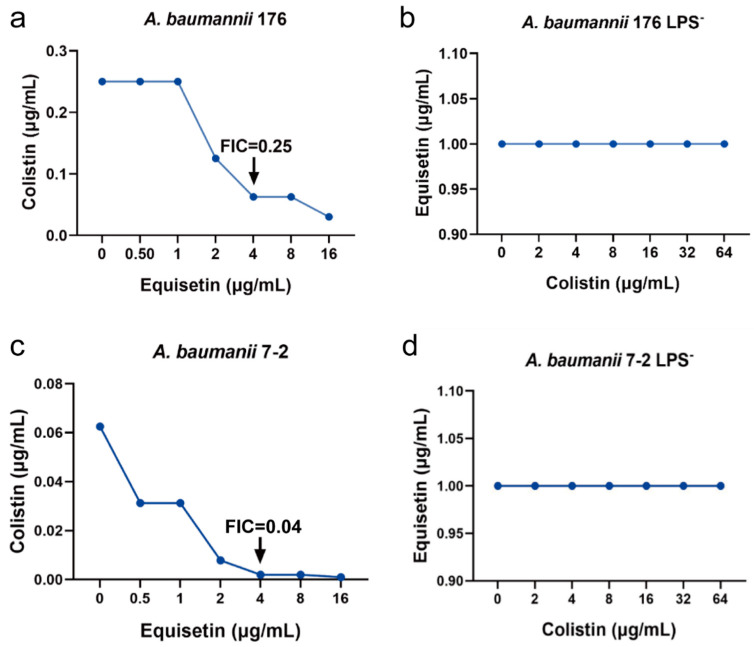
FIC for parent and LPS-deficient *A. baumannii* strains: FIC for wild type *A. baumannii* 176 (**a**) and *A. baumannii* 176 LPS^−^ (**b**); wild-type *A. baumannii* 7-2 (**c**) and *A. baumannii* 7-2 LPS^−^ (**d**).

Colistin is thought to require the insertion of the fatty-acyl chain into the OM to kill the bacteria [16]. Interestingly, the synergistic antibacterial activity can also be achieved by PBNP (Figure 2a), which lacks the fatty-acyl moiety found on colistin and is therefore incapable of inducing lysis [17]. Deacylated PMBN can sufficiently interact with and disrupt the OM, which is largely irrespective of the antibacterial mechanism [18]. Additionally, the OM disruption of bacteria and potentiation of antibiotics by PBNP and colistin are unaffected by the *mcr-1* expression [17,18]. Accordingly, we speculated that the role of colistin in drug combination is based on the destruction of the membrane. Indeed, colistin can significantly enhance the amount of other antibiotics entering the cell and exerting antibacterial synergy [19,20,21]. We used an optimized LC-MS/MS detection method to determine the intracellular accumulation of equisetin when combined with colistin or equisetin alone and found that colistin can significantly increase the accumulation of equisetin in the cell (Figure 2b). The result may indicate that equisetin enters the cytoplasm to exert its antibacterial function. Next, we used the hydrophobic fluorescent probe 1-*N*-phenylnaphthylamine (NPN) to evaluate the damaging effects of equisetin combined with colistin or equisetin and colistin alone on the OM of *E. coli* B2. As shown in Figure 3c, we found that equisetin combined with colistin showed significant damaging effects to the OM and that equisetin or colistin alone cannot damage the OM. In addition to the bacterial OM, polymyxin also plays an important role in the permeability of the bacterial cytoplasmic membrane (CM) [22]. Therefore, we used propidium iodide (PI) mediated fluorescent dyes to test the effect of the drugs on the permeability of the inner membrane. As expected, equisetin plus colistin exposure resulted in a strong PI signal from *E. coli* B2 cells (Figure 2d), indicative of the damaged CM causing the PI to fluoresce upon contact with DNA in the bacterial cytoplasm. Taken together, equisetin combined with colistin destroy the OM and CM of the bacteria. Additionally, equisetin also inhibited transcription of *mcr-1*, but not in a dose-dependent manner (Figure S2).

Since the bactericidal activity was rapid, the mechanistic basis of equisetin was considered to be associated with bacterial propagation. Thus, we evaluated whether the bacterial metabolism affects the antibacterial activity of equisetin combined with colistin and found that the combination rapidly eliminated metabolically active bacteria in 2 h (37 °C) but lost its antibacterial activity against bacteria in metabolically suppressed states (0 °C, Figure 2e). According to the rapid sterilization rate of the drug combination, we speculated that the bacteria accumulated a large number of toxic substances in a short time. Hence, we detected the content of reactive oxygen species (ROS) in bacterial cells after drug treatments. Indeed, in low levels of colistin, the accumulation of ROS in cells gradually increased with the addition of equisetin (Figure 2f).

### 2.3. FIC for Parent and LPS-Deficient A. baumannii Strains

To further explore the synergistic mechanism of equisetin and colistin, we studied two LPS-deficient *A. baumannii* strains that are inactivated in the lipid A biosynthesis [23,24]. We first determined the MIC of equisetin and colistin on wild-type and LPS-deficient *A. baumannii* isolates and found that colistin resistance increased more than 100-fold, whereas equisetin resistance decreased more than 100-fold relative to the wild-type parent *A. baumannii* (Table 2). Our results demonstrated that LPS deficiency of strains fostered resistance to colistin but was sensitive to equisetin, which is exclusively active against Gram-positive bacteria. Furthermore, equisetin lacked synergy with colistin on LPS-deficient *A. baumannii*, contrary to the wild type (Figure 3). These findings denote that outer-membrane LPS are the major obstacle to equisetin-dependent killing in *A. baumannii*. 

### 2.4. In Vivo Efficacy of Equisetin Combined with Colistin

**Figure 4:**
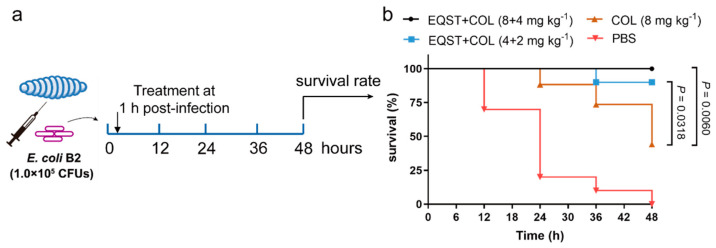
Efficacy of equisetin combined with colistin in *G. mellonella* infection model. (**a**) Scheme of the experimental protocol for the *G. mellonella* infection model; (**b**) survival rates of *G. mellonella* larva infected with *E. coli* B2 (1.0 × 10^5^ CFUs) in the presence of equisetin combined with colistin at 8 mg kg^−1^ + 4 mg kg^−1^ and 4 mg kg^−1^ + 2 mg kg^−1^ (*n* = 10). Colistin at 8 mg kg^−1^ was used as a positive control. *p* values were determined using a two-sided, Mann–Whitney *U*-test.

The encouraging antibacterial activity of equisetin combined with colistin in vitro inspired us to further investigate their therapeutic potential in vivo. We constructed a resistant bacteria infection model of *E. coli* B2 in *G. mellonella* larvae, as was previously reported [19]. After 1 h post-infection, equisetin combined with colistin was supplied with different dose gradients (Figure 4a). The experimental results showed that equisetin combined with colistin at 8 mg kg^−1^ + 4 mg kg^−1^ fully protected *G. mellonella* from infection, which suggests that equisetin combined with colistin is a promising solution to treat MDR-associated infections in vivo (Figure 4b).

## 3. Discussion

We are in a “post-antibiotic era” and there are few clinical antibacterial candidates, especially for Gram-negative pathogens [25]. The OM of Gram-negative bacteria is an effective barrier in the outer leaflet that protects bacteria from toxic environmental insults, including antibiotics. Given the long development duration of antibacterial drugs and the high probability of failure, we must preserve the existing drugs. Combination therapy with existing drugs is a key to combat MDR infections and extend their shelf lives [26]. Potentiators that restore the activity of current antibiotics can expand the antibacterial spectrum as well. For example, the inhibitors of β-lactamases and sulfonamide synergists overcome a number of pathogens [27]. Notably, colistin disrupts the OM of Gram-negative bacteria to permit other compounds access to their cytoplasmic targets [28]. Thus, combinations of colistin with hydrophobic antibiotics [29] or adjuvants [30] are often used in the treatment of MDR Gram-negative infections. Colistin binding to the negatively charged lipid A phosphate groups of an LPS component causes membrane destabilization, leading to the increase of cell envelope permeability, leakage of cellular contents and ultimately cell death [18]. Despite that the medication of phosphate groups decreases the therapeutic utility of colistin, colistin still can paralyze the integrity of colistin-resistant bacteria [31,32]. For instance, colistin in combination with rifampicin [33], macrolides [34], tigecycline [35], carbapenems and glycopeptides [36] achieves promising therapeutic outcomes. Indeed, although hydrophobic antibiotics are conventionally active against Gram-positive bacteria such as rifampicin and macrolides, they have shown favorable prospects against Gram-negative pathogens in the presence of colistin or other Gram-negative OM disrupting compounds [37].

Compared to natural antibiotics of terrestrial origins, marine organisms that survive in high salinity, high pressure, low temperature and oligotrophic environments become appealing for the discovery of new compounds. Marine fungi derived from sponges are outstanding sources of natural products with various biological activities [38]. Equisetin isolated from a marine sponge-associated fungal strain *Fusarium* can kill diverse MDR Gram-positive bacteria without cross-resistance and detectable resistance [15,39]. In addition to effective antimicrobial effects, equisetin also has activity against HIV-1 integrasel [40] and mitochondria [41]. In combination with colistin, equisetin displays antibacterial activity against Gram-negative pathogens including colistin-resistant bacteria, enhancing colistin activity substantially and reducing its cytotoxicity accordingly.

Interestingly, the rapid bactericidal ability of equisetin combined with colistin against *E. coli* (Figure 1d) is similar to the equisetin-mediated killing curve of *S. aureus* [15]. Moreover, the result that LPS-deficient Gram-negative bacteria are sensitive to equisetin indicates that equisetin may target the same component in both Gram-positive and Gram-negative bacteria. Previous studies suggest that the formation of ROS is a common mechanism of bacterial death induced by bactericidal antibiotics [42,43,44]. Accumulated ROS break DNA, peroxidate lipids, carbonylate proteins and so on [45]. Correspondingly, ROS-mediated damages accelerate ROS accumulation, leading to metabolic perturbation and cell death [46]. Equisetin shows an ROS-independent bactericidal mechanism (data unpublished). Thus, we speculate that colistin facilitates the entry of equisetin to the MDR Gram-negative bacteria, while colistin promotes intracellular ROS accumulation that contributes to rapid bacterial killing. Additionally, combination antibiotic therapy is frequently used to treat severe Gram-negative infections in clinics [47]. Equisetin combined with colistin is highly effective in eradicating MDR Gram-negative bacteria in *G. mellonella* infection models. Altogether, these findings suggest that equisetin exhibits a distinct mode of action against bacteria and possesses promising pharmacokinetic properties.

In summary, our study shows that equisetin acts synergistically with colistin to kill various MDR Gram-negative bacteria (including colistin-resistant strains) and restores the efficacy of colistin in vivo. We find drug combinations alter the permeability of the OM and CM of Gram-negative bacteria, permitting the increased accumulation of intracellular equisetin to exert antibacterial activity. Collectively, we demonstrate that equisetin combined with colistin represents an attractive strategy for the treatment of infections caused by MDR Gram-negative pathogens.

## 4. Materials and Methods

### 4.1. Bacterial Isolates

The standard strains in this study were purchased from China General Microbial Collection and Management Center (CGMCC) and the other clinically resistant strains were isolated by our laboratory, including *E. coli* B2 and 23 *mcr-1* positive bacteria [19].

### 4.2. Drugs

Ampicillin, ceftriaxone, meropenem, erythromycin, tilmicosin, kanamycin, gentamycin, ofloxacin, norfloxacin, rifampicin, tigecycline, tetracyclines, florfenicol and colistin were purchased from the China Institute of Veterinary Drugs Control (IVDC). Equisetin was isolated from the fungus *Fusarium equiseti* 33-10 [15].

### 4.3. Antibacterial Test

The minimum inhibitory concentrations (MICs) of equisetin and colistin were determined by the standard broth microdilution method, according to the CLSI 2021 guideline. Briefly, equisetin or colistin were mixed with equal volumes of bacterial suspensions in Mueller–Hinton broth (MHB) containing approximately 1.5 × 10^6^ colony-forming units (CFUs)/mL in a clear UV-sterilized 96-well microtiter plate. After 16–20 h incubation at 37 °C, the MIC values were defined as the lowest concentrations of antibiotics with no visible growth of bacteria.

### 4.4. FIC Index Determination

FIC index (FICI) was determined using chequerboard assays. Colistin was diluted along the ordinate, while equisetin was diluted along the abscissa by MHB in a 96-well microtiter plate in a final volume of 100 µL. Then, bacterial suspensions were diluted by approximately 1.5 × 10^6^ CFUs/mL in MHB and 100 µL were added to 96-well microtiter plates containing antibiotics. After 16–20 h of incubation at 37 °C, the FICI was calculated according to the following formula:

FIC index = MIC_AB_/MIC_A_ + MIC_BA_/MIC_B_ = FIC_A_ + FIC_B_

MIC_A_ is the MIC of compound A alone, MIC_AB_ is the MIC of compound A in combination with compound B, MIC_B_ is the MIC of compound B alone, MIC_BA_ is the MIC of compound B in combination with compound A; FIC_A_ is the FIC of compound A and FIC_B_ is the FIC of compound B. Synergy is defined as an FIC index ≤ 0.5.

### 4.5. Growth Curves of Bacteria

The overnight cultures of *E. coli* B2 were standardized to match a 0.5 McFarland turbidity standard and followed by 1:100 dilution in MHB. Different concentrations of equisetin, colistin or the combination of equisetin with colistin were added to a 96-well microplate and mixed with an equal volume of culture diluted 1:100 in MHB with the plate lid covered. The growth curves were recorded under the wavelength of 600 nm with an interval of 4 h at 37 °C, using an Infinite M200 Microplate reader (Tecan) in the long-term model. PBS was used as a negative control in the experiment.

### 4.6. Time-Kill Assays

*E. coli* B2 was cultured to the exponential phase at 37 °C with shaking at 200 revolutions per minute (rpm) for 2 h and then diluted in MHB to a desired concentration at 10^6^ to 10^7^ CFU mL^−1^. Then, bacteria were treated by equisetin, colistin or the combination of equisetin with colistin in culture tubes at 37 °C with shaking at 200 rpm. Subsequently, 10-fold serially diluted suspensions were plated on Tryptose Soya Agar (TSA) plates and incubated at 37 °C overnight for enumeration of bacterial colonies. PBS was used as a negative control in the experiment.

### 4.7. Antibiotic Accumulation Analysis

The accumulation of equisetin in *E. coli* B2 was determined based on LC–MS/MS analysis. Mobile phase A: 0.1% formic acid aqueous solution; mobile phase B: 0.1% formic acid methanol solution; chromatographic column: C18 column (2.1 × 100 mm, 1.7 μm); flow rate is 0.3 mL/min; gradient elution ratio is as follows: 0.1–1.0 min, 60% A; 1.0–3.0 min, 95% A; 3.0–5.0 min, 95% A; 6.0–8.0 min, 60% A; injection volume is 2 µL. Multiple reaction monitoring (MRM) in the positive ion mode was used to quantify the content of intracellular equisetin (Table S3, Figure S1).

### 4.8. Outer Membrane Permeability Assay

The fluorescent probe NPN was used to evaluate the outer membrane integrity of *E. coli* B2 when treated by different concentrations of equisetin combined with colistin or with drugs alone. Briefly, *E. coli* B2 grown overnight at 37 °C were washed and resuspended in 5 mmol/L of HEPES (pH 7.2, +5 mmol/L of glucose) to obtain an OD_600_ of 0.5. NPN was added to a final concentration of 10 µmol/L and the mixture was incubated at 37 °C for 30 min. A total of 190 µL of probe-labelled bacterial cells were added to a 96-well plate and then 10 µL of the drug combination or the drug alone was added. After incubating for 30 min, fluorescence was measured on an Infinite M200 Microplate reader (Tecan) with the excitation wavelength at 350 nm and the emission wavelength at 420 nm.

### 4.9. Cytoplasmic Membrane Permeability Assay

*E. coli* B2 grown overnight at 37 °C were washed and resuspended in 0.01 mol/L of PBS (pH 7.4) to obtain an OD_600_ of 0.5. PI was added to a final concentration of 10 µmol/L and the mixture was incubated at 37 °C for 10 min. Then, after being treated by the drug combination or drug alone, fluorescence was measured with the excitation wavelength at 535 nm and emission wavelength at 615 nm.

### 4.10. ROS Measurement

The levels of ROS in *E. coli* B2, when treated by different concentrations of equisetin combined with colistin, were measured with 10 µmol/L of 2′,7′-dichlorofluorescein diacetate (DCFH-DA), following the manufacturer’s instructions (Beyotime, catalogue No. S0033). Briefly, *E. coli* B2 grown overnight at 37 °C were washed and resuspended in 0.01 mol/L of PBS (pH 7.4) to obtain an OD_600_ of 0.5. DCFH-DA was added to a final concentration of 10 µmol/L and the mixture was incubated at 37 °C for 30 min. After washing with 0.01 mol/L of PBS three times, 190 µL of probe-labelled bacterial cells were added to a 96-well plate before adding 10 µL of 1 µg/mL of colistin combined with different concentrations of equisetin. After incubating for another 30 min, fluorescence intensity was immediately measured with the excitation wavelength at 488 nm and the emission wavelength at 525 nm using the Infinite M200 Microplate reader (Tecan).

### 4.11. Reverse Transcription-(RT)PCR Analysis

*E. coli* B2 was grown overnight at 37 °C and treated with equisetin (0–64 µg/mL). Total RNA was extracted using the EASYspin Plus kit (Aidlab, catalogue no. RN0801) and quantified by the ratio of absorbance (260 nm:280 nm) using a Nanodrop spectrophotometer (Thermo Fisher Scientific, Waltham, American). Before the complementary DNA synthesis, the RNA from all bacterial cells was adjusted to an identical concentration of 1 µg and a reverse transcription of extracted RNA was performed using the PrimeScript RT reagent Kit (Takara, catalogue no. RR047A), following the manufacturer’s protocol. 

The levels of *mcr-1* in treated *E. coli* groups were determined by RT-PCR relative to the 16S rRNA mRNA levels of the control gene. According to the previously reported primers for 16S rRNA and *mcr-1* [35], RT-PCR was performed with SYBR Green qPCR kit (TaKaRa). Thermal cycling was performed by a standard two-step PCR amplification method, 95 °C for 30 s, 40 cycles of 95 °C for 5 s and 60 °C for 34 s. The ABI quantstudio TM 7 detection system (Applied Biosystem) for RT-PCR detection and a 2^−ΔΔCt^ method was used to determine the fold change of gene expression.

### 4.12. G. mellonella Infection Model

The synergy between equisetin and colistin was accessed in the *G. mellonella* infection model. The larvae of *G. mellonella* (purchased from Tianjin Huiyude Biotech Company) were randomly divided into four groups (*n* = 10 per group) and infected with 10 µL of *E. coli* B2 suspension (1.0 × 10^5^ CFUs) at the right posterior gastropoda. After 1 h post-infection, *G. mellonella* was treated with PBS, colistin (8 mg kg^−1^) or the combination of equisetin with colistin (8 mg kg^−1^ + 4 mg kg^−1^ and 4 mg kg^−1^ + 2 mg kg^−1^) at the left posterior gastropoda. Survival rates of *G. mellonella* were recorded over 48 h. PBS was used as negative control and colistin at 8 mg kg^−1^ was used as a positive control in the experiment.

## 5. Conclusions

In conclusion, these results demonstrate that equisetin combined with colistin shows great efficacy for treating MDR Gram-negative bacteria infections in vitro and in vivo, which suggests that drug combination holds promise for overcoming antibacterial resistance. Therefore, given the fact that development of new antibiotics declines rapidly, drug combination can be a promising approach to extend the life of the already existing antibacterial drug arsenal.

## Figures and Tables

**Table 1:** The activity of equisetin combined with colistin against 23 *mcr-1* positive isolates.

Equisetin + Colistin (µg/mL)	Inhibition Ratio
1 + 1	73.91% (17/23)
2 + 0.5	82.60% (19/23)
2 + 1	91.30% (21/23)
4 + 0.5	95.65% (22/23)
4 + 1	100.00% (23/23)

**Table 2:** MICs of parent and LPS-deficient *A. baumannii* strains.

*A. baumannii* Strain	Colistin (µg/mL)	Equisetin (µg/mL)
176 (wild type)	0.25	>128
176 (mutant lacking LPS)	>128	1
7-2 (wild type)	0.125	>128
7-2 (mutant lacking LPS)	>128	1

## Supplementary Materials

The following are available online at https://www.mdpi.com/article/10.3390/antibiotics10101263/s1, Table S1: The FICI of antibiotics combined with equisetin against *E. coli* B2, Table S2. Resistant isolates used in this study. Table S3: MRM parameters for the determination of equisetin by LC-MS/MS, Figure S1: Precursor ion mass spectrum of equisetin standard solutions (1 μg/mL). Figure S2: Equisetin inhibits transcription of *mcr-1* determined by reverse transcription (RT)-PCR.
