# Supplementary material for: Equisetin Restores Colistin Sensitivity against Multi-Drug Resistant Gram-Negative Bacteria
Source: Antibiotics (Basel). 2021 Oct 18;10(10):1263. doi: 10.3390/antibiotics10101263 (PMC8532683; doi:10.3390/antibiotics10101263)
Supplement: Supplementary file 1 [file antibiotics-10-01263-s001.zip › antibiotics-1377501-SI.pdf]

Supplement Materials:

Table S1. The FICI of antibiotics combined with equisetin against *E. coli* B2.

Antibiotic	MIC of Antibiotic	FIC_A	MIC of Equisetin[1]	FIC_E	FICI
Colistin	8	0.125	1	0.0001	0.125
Rifampin	>128	0.5	16	0.5	1
Ofloxacin	32	0.5	8	0.5	1
Erythromycin	>128	0.5	16	0.5	1
Tilmicosin	16	0.5	4	0.5	1
Tetracyclines	>128	0.5	16	0.5	1
Kanamycin	>128	0.5	16	0.5	1
Norfloxacin	32	0.5	8	0.5	1
Florfenicol	>128	0.5	16	0.5	1
Gentamycin	32	0.5	8	0.5	1
Meropenem	4	0.5	4	0.5	1
Ceftriaxone	128	0.5	16	0.5	1
Ampicillin	>256	0.5	16	0.5	1
Tigecycline	0.25	0.5	4	0.5	1

[1]In the presence of sub-MIC concentrations of antibiotics, the MIC of equisetin against *E. coli* B2.
Fractional inhibitory concentration (FIC) indices were calculated based on chequerboard broth microdilution assays. FIC index (FICI) is the sum of FIC_A and FIC_E. FIC_A is the FIC of tested antibiotic. FIC_E is the FIC of equisetin.

Table S2. Resistant isolates used in this study.

Strains	Description	Reference
Escherichia coli 10	*mcr-1*	This study
E. coli 29	*mcr-1*	This study
E. coli 206	*mcr-1*	This study
E. coli 213	*mcr-1*	This study
E. coli 250	*mcr-1*	This study
E. coli 258	*mcr-1*	This study
E. coli B2	bla_{NDM-5} + *mcr-1*	[1]
E. coli 267	*mcr-1*	This study
E. coli 294	*mcr-1*	This study
E. coli 296	*mcr-1*	This study
E. coli 299	*mcr-1*	This study
E. coli 162	*mcr-1*	This study
E. coli 315	*mcr-1*	This study
E. coli 378	*mcr-1*	This study
E. coli 393	*mcr-1*	This study
E. coli 638	*mcr-1*	This study
E. coli 878	*mcr-1*	This study
E. coli 1323	*mcr-1*	This study
E. coli 1334	*mcr-1*	This study
E. coli 1336	*mcr-1*	This study
E. coli 1794	*mcr-1*	This study
E. coli 12120478	*mcr-1*	This study
E. coli WZ3909	*mcr-1*	This study
Klebsiella pneumoniae 91	*mcr-1*	This study
Enterobacter cloacae 16-15	*mcr-1*	This study
Raoultella planticola 16-15	*mcr-1*	This study
Providencia alcalifaciens 16-1	*mcr-1*	This study
Serratia marcescens 16-99	*mcr-1*	This study

Raoultella ornithinolytica 16-68	*mcr-1*	This study
Acinetobacter veronii 172	*mcr-3*	[2]
Acinetobacter baumannii 2-38	*mcr-1*	This study
Salmonella enterica 8H-3	*mcr-1*	This study

Table S3. MRM parameters for the determination of equisetin by LC-MS/MS

Precursor ion (*m*/*z*)	Product ions (*m*/*z*)	Q1 pre bias (V)	Collision energy (eV)	Q3 pre bias (V)
374.30	175.20	-19.0	-18.0	-19.0
	170.10	-11.0	-24.0	-18.0

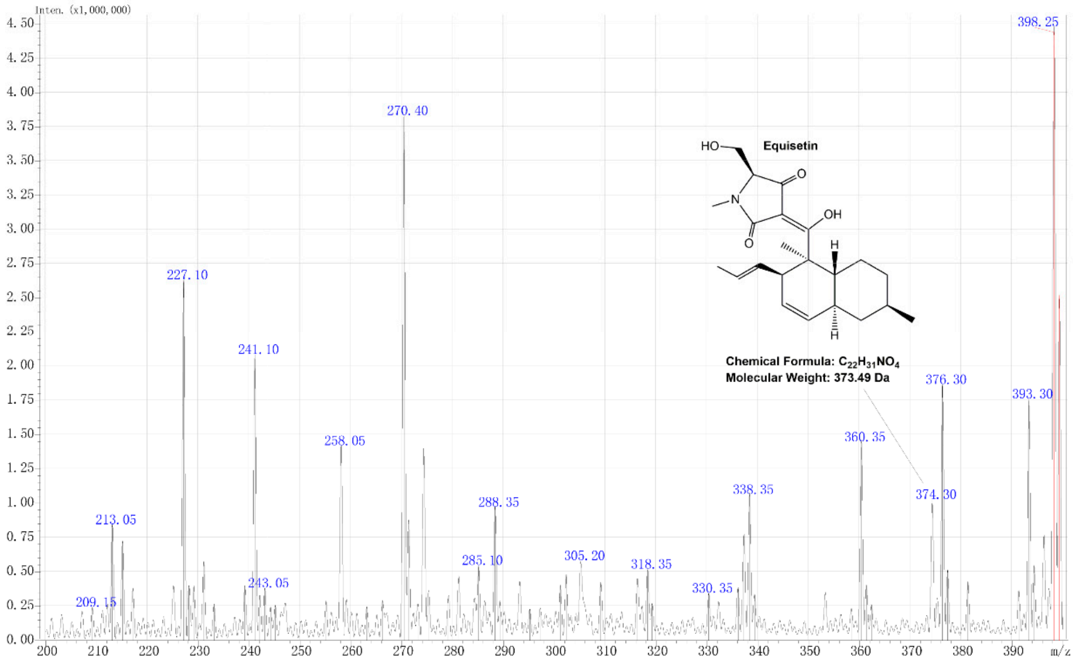

Figure S1. Precursor ion mass spectrum of equisetin standard solutions (1 μg/mL).

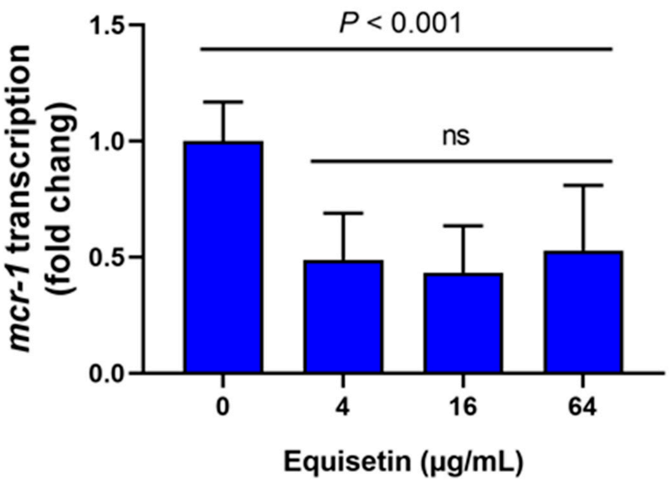

Figure S2. Equisetin inhibits transcription of *mcr-1* determined by reverse transcription (RT)-PCR. Data are representative of three independent experiments, and the mean of three biological

replicates is shown and error bars represent the s.d. *P* values were determined using an unpaired, two-tailed Student's t-test.

References

1. Song, M.; Liu, Y.; Huang, X.; Ding, S.; Wang, Y.; Shen, J.; Zhu, K. A broad-spectrum antibiotic adjuvant reverses multidrug-resistant Gram-negative pathogens. *Nat. Microbiol.* **2020**, doi:10.1038/s41564-020-0723-z.
2. Ling, Z.; Yin, W.; Li, H.; Zhang, Q.; Wang, X.; Wang, Z.; Ke, Y.; Wang, Y.; Shen, J. Chromosome-Mediated mcr-3 Variants in Aeromonas veronii from Chicken Meat. *Antimicrob. Agents. Ch.* **2017**, *61*, doi:10.1128/aac.01272-17.
